# Daily Sedation Interruption Versus Continuous Sedation in Mechanically Ventilated Pediatric Patients: A Systematic Review and Meta-Analysis of Randomized Controlled Trials

**DOI:** 10.7759/cureus.101727

**Published:** 2026-01-17

**Authors:** Marwa H Alhag, Hadeel M Awami, Lugain Samman, Bandar S Alshreef, Alaa Ahmed, Mohamed A Beda, Assraf Abu Ayish, Sarah K Hussein, Samar Mansoor, Raghad Alkhulaidi, Tasneem Ashraf, Sarah Marzouk, Redwan M Mulla, Shahad A Aloufi

**Affiliations:** 1 Pediatric Emergency, Maternity and Children's Hospital (MCH), Hail, SAU; 2 Pediatrics, College of Medicine and Surgery, King Abdulaziz University, Jeddah, SAU; 3 Healthcare Management, College of Applied Medical Sciences, Shaqra University, Shaqra, SAU; 4 General Practice, Sudan International University, Khartoum, SDN; 5 Pediatric Intensive Care Unit, Madinah Cardiac Center, Madinah, SAU; 6 Pediatric Intensive Care Unit, Barzilai Medical Center, Ashkelon, ISR; 7 General Pediatrics, Tameside General Hospital, Manchester, GBR; 8 Medicine, Ibn Sina National College for Medical Studies, Jeddah, SAU; 9 General Pediatrics, King Abdullah Specialized Children's Hospital, Jeddah, SAU; 10 Pediatrics, College of Medicine, Taibah University, Madinah, SAU; 11 Pediatrics, Ministry of Health, Saudi Arabia, Mecca, SAU

**Keywords:** daily sedation interruption, mechanical ventilation, meta-analysis, pediatric intensive care unit, randomized controlled trial, sedation protocol

## Abstract

Sedation management in critically ill children is challenging, with continuous infusions often leading to oversedation, prolonged mechanical ventilation, and iatrogenic withdrawal syndrome. Daily sedation interruption (DSI) is a strategy to mitigate these risks, but evidence regarding its efficacy and safety in the pediatric population is conflicting. This systematic review and meta-analysis aimed to evaluate the impact of DSI versus continuous sedation on clinical outcomes in mechanically ventilated pediatric patients. A systematic search of PubMed, Embase, and CENTRAL was conducted to identify randomized controlled trials (RCTs) comparing DSI with continuous sedation in children (0-18 years). The primary outcome was the duration of mechanical ventilation. Secondary outcomes included length of stay (LOS) in the pediatric ICU (PICU), total drug exposure, sedation depth, and adverse events. Data were pooled using a random-effects model. Trial sequential analysis (TSA) was performed to assess the sufficiency of the evidence, and the certainty of evidence was graded using the Grading of Recommendations Assessment, Development and Evaluation (GRADE) methodology. Six RCTs comprising 2,810 patients were included. In the pooled analysis, DSI was associated with a reduction in the duration of mechanical ventilation (mean difference (MD) -1.01 days; 95% CI -2.07 to 0.05; p = 0.06) and PICU LOS (MD -0.85 days; p = 0.31), though neither reached statistical significance. Significant heterogeneity was observed (I²= 51.8%). Subgroup analysis revealed that DSI reduced ventilation time in studies with a nonstandardized control group (MD -1.85 days) but offered no benefit over protocolized sedation (MD -0.41 days). No significant differences were found in mortality (risk ratio (RR) 1.08), accidental extubation (RR 0.89), or re-intubation rates (RR 0.85). TSA indicated that the current evidence is inconclusive, having not met the required information size to detect a 1.5-day reduction in ventilation. DSI is safe in critically ill children but does not consistently reduce the duration of mechanical ventilation compared to modern protocolized sedation care. Its utility may be greatest in resource-limited settings lacking robust sedation titration protocols. Given the low certainty of current evidence, DSI should be considered a component of multimodal sedation management rather than a standalone standard of care.

## Introduction and background

Sedation and analgesia are integral components of the management of critically ill pediatric patients, serving to alleviate pain, reduce anxiety, and facilitate tolerance of life-sustaining interventions such as mechanical ventilation [1,2]. However, achieving optimal sedation is a complex clinical challenge, as the practice of continuous sedative infusion is effective for maintaining comfort, but it is associated with drug accumulation, tolerance, and iatrogenic withdrawal syndrome [3]. Evidence suggests that sedation practices in the pediatric ICU (PICU) are often suboptimal, with oversedation reported in up to 40-60% of ventilated children [2,4]. Excessive sedation is associated with adverse clinical outcomes, including prolonged mechanical ventilation, increased length of ICU stay, and a higher incidence of ventilator-associated pneumonia [2,5], while under-sedation may precipitate physiological instability, unplanned extubation, and long-term psychological sequelae [1,3].

Strategies such as protocolized titration and daily sedation interruption (DSI) have been proposed to mitigate the complications of continuous sedation. Unlike protocolized sedation, which depends on continuous algorithmic adjustment by bedside nurses to maintain a target score, DSI functions as a daily systemic reset. Operationalized in practice, DSI involves the planned cessation of sedative infusions, contingent on specific safety criteria regarding hemodynamic and respiratory stability, until the patient awakens or follows commands. Once the patient awakens or if significant distress occurs, infusions are typically resumed at a reduced dose (e.g., 50% of the previous rate), which allows for the clearance of accumulated metabolites and a more accurate assessment of neurological status [5,6]. DSI reduces the duration of mechanical ventilation and ICU stay in adults; however, its efficacy and safety in the pediatric population remain controversial [4,5].

Pediatric patients present unique physiological and developmental challenges compared to adults, including different pharmacokinetic profiles and a higher propensity for agitation upon awakening [3,7]. Previous studies evaluating DSI in children have yielded conflicting results, with some trials suggesting significant benefits in reducing ventilation time [8,9], while others, including a large multicenter cluster randomized trial, found no advantage of protocolized sedation with or without DSI over usual care [4,10]. In addition, the safety profile of DSI in children, specifically regarding the risks of accidental extubation and hemodynamic instability, remains a critical concern for clinicians [2,6].

Given the heterogeneity of existing evidence and the lack of a standardized consensus, a systematic evaluation of the available data is needed. Therefore, this systematic review and meta-analysis aimed to assess the efficacy and safety of DSI compared with continuous sedation in mechanically ventilated pediatric patients. Specifically, the impact of DSI on the duration of mechanical ventilation, length of stay (LOS), total drug exposure, and incidence of adverse events was evaluated while accounting for methodological variations, such as baseline sedation protocols and study design.

## Review

Methods

Protocol and Registration

This systematic review and meta-analysis was conducted in accordance with the Preferred Reporting Items for Systematic reviews and Meta-Analyses (PRISMA) guidelines [11]. The protocol was established a priori to define the inclusion criteria, search strategy, and statistical analysis (PROSPERO; CRD420251170984).

Data Sources and Search Strategy

A systematic search was performed across electronic databases, including PubMed/MEDLINE, Embase, and the Cochrane Central Register of Controlled Trials (CENTRAL), from inception to December 2025. The search strategy used a combination of Medical Subject Headings (MeSH) and free-text keywords related to “pediatric”, “intensive care”, “mechanical ventilation”, “sedation interruption”, and “sedation holidays”. There were no language restrictions. The reference lists of the included trials and relevant review articles were hand-searched to identify additional eligible studies.

Eligibility Criteria

Studies were selected based on the PICO framework, as the population was pediatric patients (aged 0-18 years) receiving mechanical ventilation in a PICU. DSI is defined as the planned daily cessation or minimization of sedative infusions until the patient awakens or follows commands. Continuous sedation administered either as standard care (manual titration) or via a protocolized sedation algorithm without daily interruption was used as the comparator. The primary outcome was the duration of mechanical ventilation, while the secondary outcomes included PICU LOS, total cumulative drug dose, sedation depth, and adverse events (mortality, accidental extubation, and re-intubation).

Data Extraction and Quality Assessment

Two independent reviewers screened the titles, abstracts, and full-text articles. Data extraction was performed using a standardized form capturing the study design, sample size, patient demographics, sedation protocols, and outcome data.

The methodological quality of the included randomized controlled trials (RCTs) was assessed using the Cochrane Risk of Bias 2 (RoB 2) tool [12]. Studies were graded as low risk, some concerns, or high risk across five domains: randomization process, deviations from intended interventions, missing outcome data, measurement of the outcome, and selection of the reported results.

Statistical Analysis

All statistical analyses were performed using R statistical software (version 4.5.1) with the meta, metafor, and RTSA packages. For continuous outcomes (e.g., duration of ventilation), the mean difference (MD) was calculated using the inverse-variance method. For studies reporting nonparametric data (medians and IQRs), means and SDs were estimated using the methods described by Wan et al. [13] and Luo et al. [14] to facilitate data pooling [12].

For binary outcomes (e.g., mortality), the risk ratio (RR) with 95% CIs was used. To assess sedation depth across studies using disparate scales (e.g., Ramsay vs. COMFORT-B), the standardized MD (SMD, Hedges’ g) was calculated [15].

Data from cluster randomized trials were adjusted to prevent unit-of-analysis errors and artificial inflation of statistical weight using the design effect (Deff). The effective sample size (Neff) was calculated as

$\frac{N}{1 + (M - 1) \times \mathrm{ICC}},$

where M is the average cluster size, and ICC is the intracluster correlation coefficient reported in the primary trial [12].

Due to the anticipated clinical and methodological diversity among the included studies (e.g., variations in control group protocols), a random-effects model (DerSimonian-Laird) was employed for all analyses. This estimator was selected as the standard method for estimating between-study variance in clinical meta-analyses to provide a conservative estimate of the effect size [15].

Statistical heterogeneity was assessed using Cochran’s Q test and quantified using the I² statistic. An I² value greater than 50% was considered indicative of substantial heterogeneity [12]. Sources of heterogeneity were explored using subgroup analyses stratified by the control group strategy (manual vs. protocolized) and random-effects meta-regression for continuous moderators (e.g., mean patient age).

Assessment of Reporting Biases

Publication bias and small study effects were assessed visually using funnel plots [16]. Statistical asymmetry was evaluated using Egger’s linear regression test [17]. The potential for bias induced by incompletely reported outcomes in individual studies [18] was acknowledged and visualized using an outcome-reporting matrix. Although selection method approaches [19] and trim-and-fill methods [20] are often used to adjust for missing studies, they were not applied in the primary analysis because of the limited number of included studies (k < 10), which reduces the power of these correction techniques [21,22].

Trial Sequential Analysis (TSA)

TSA was performed to control the risk of Type I errors due to repetitive testing and sparse data. Monitoring boundaries were constructed for the primary outcome based on a priori defined parameters: a two-sided α of 5%, power of 80%, and a clinically meaningful minimal clinical difference (MCD) of 1.5 days. The required information size (RIS) was calculated to determine whether the current evidence base was sufficiently powered [23,24].

Certainty of Evidence

The overall certainty of the evidence was graded using the Grading of Recommendations Assessment, Development and Evaluation (GRADE) approach. The evidence was downgraded based on limitations in the study design (risk of bias), inconsistency of results, indirectness of evidence, imprecision, and publication bias [12].

Results

Search Results and Study Selection

The initial electronic search across the designated databases identified 3439 citations. After removing duplicates and performing an initial screening based on titles and abstracts, 56 records remained for full-text assessment. After a detailed review of the full texts against the prespecified inclusion and exclusion criteria, 50 articles were excluded. The primary reasons for exclusion were an ineligible study design, adult population, incorrect intervention, or lack of specific outcomes.

**Figure 1:**
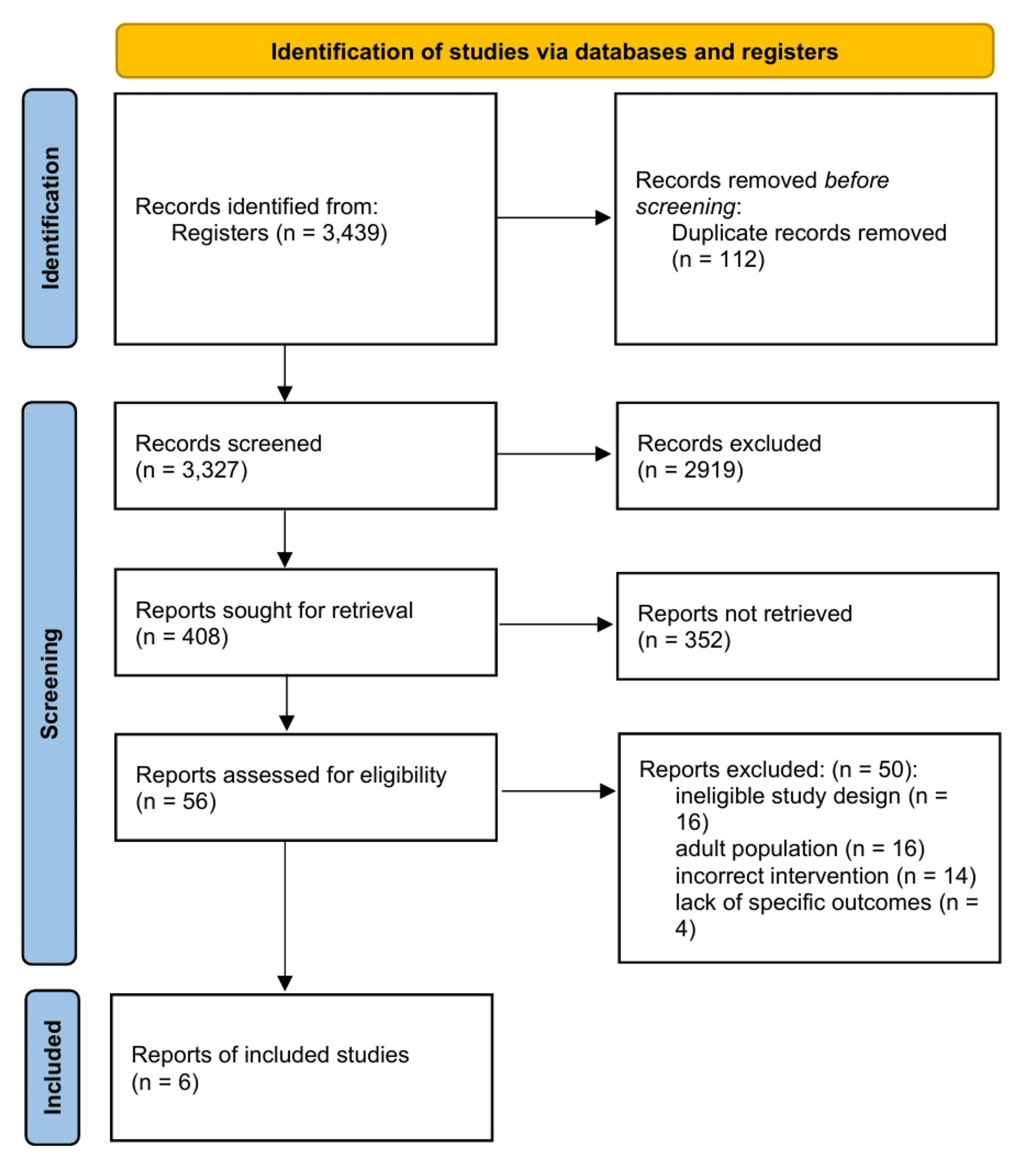
PRISMA flow diagram PRISMA, Preferred Reporting Items for Systematic reviews and Meta-Analyses

Six RCTs met the eligibility criteria and were included in the systematic review and meta-analysis [4,8-10,25,26]. The selection process is illustrated in the PRISMA flow diagram (Figure 1).

Study Characteristics

The six included studies comprised a total of 2,810 randomized pediatric patients. The sample sizes ranged from 30 patients in the pilot study by Verlaat et al. [26] to 2,449 patients in the large-scale RESTORE trial by Curley et al. [10]. The studies were conducted in diverse settings, including the Netherlands [4,26], the United States [10,25], India [8], and Indonesia [9]. The aggregate population spanned a broad age range, from infants (median age ~2 months) [4,26] to older children (mean age ~4.7 years) [10].

Regarding the study design, five studies used a standard individual-patient randomization design, while one study [10] employed a cluster randomized factorial design involving 31 PICUs. To account for the clustering effect and avoid unit-of-analysis errors, the effective sample size for this study was statistically adjusted using the reported ICC (0.046) to derive Neff prior to data pooling.

The specific clinical indications for mechanical ventilation varied across trials, as five studies included a general mix of medical and surgical PICU patients, with respiratory failure being the predominant admission diagnosis, while one study [25] enrolled patients following pediatric cardiothoracic surgery, introducing a source of clinical heterogeneity regarding the trajectory of recovery and sedation requirements.

Validated scales were used to target sedation levels, including the COMFORT-B scale [4, 26], Ramsay Sedation Scale [8], and FLACC scale [25]. In addition, there was notable heterogeneity in the control group protocols, as two studies [8,9] compared DSI against a control group receiving continuous sedation without a standardized titration protocol (“Manual/ad-hoc” baseline), while three studies [4,10,26] compared DSI against a control group managed with a protocolized sedation algorithm (“Protocolized” baseline). Penk et al. [25] used a distinct design comparing an “intermittent dosing only” strategy against “continuous infusion plus intermittent dosing.”

The detailed characteristics of the included studies, including intervention protocols, sedation scales, and primary endpoints, are presented in Table 1.

**Table 1:** Characteristics of included RCTs ^*^ Sample size represents raw patient count; effective sample size adjusted for clustering in meta-analysis. DSI, daily sedation interruption; LOS, length of stay; MV, mechanical ventilation; PICU, pediatric ICU; RCT, randomized controlled trial; SBS, State Behavioral Scale

Study	Country	Design	Sample size (N)	Population	Intervention (DSI) protocol	Control protocol	Sedation scale	Primary outcome
Vet et al. [4]	Netherlands	Multicenter RCT	N = 129 (DSI = 66, Cont = 63)	3 PICUs; MV expected >48 hours; age 0-18 years	Blinded DSI + protocolized sedation. Daily screen. Restart at 50% dose.	Protocolized sedation only (blinded continuous infusion)	COMFORT-B	Ventilator-free days at D28
Gupta et al. [8]	India	RCT	N = 102 (DSI = 46, Cont = 56)	PICU; MV >48 hours; Age 1 month-12 years	Daily interruption at 8:00 AM until awake/agitated. Restart at 50% dose.	Continuous infusion (midazolam + morphine) titrated to Ramsay 3-4.	Ramsay	Duration of MV, PICU LOS
Azis et al. [9]	Indonesia	RCT	N = 40 (DSI = 22, Cont = 18)	PICU; MV >24 hours; Age 1 month-18 years	Daily interruption after 24 hours. Restart at 50% dose if distressed.	Continuous sedative infusion (midazolam) titrated to COMFORT 11-22.	COMFORT	Duration of MV
Curley et al. [10]	USA	Cluster RCT	N = 2449^*^ (Protocol = 1225, Usual = 1224)	31 PICUs; Acute resp. failure; Age 2 weeks-17 years	Nurse-implemented protocol: Targeted sedation, arousal assessments, and weaning.	Usual care (physician discretion)	SBS, WAT-1	Duration of MV
Penk et al. [25]	USA	RCT	N = 60 (Intermittent = 30, Cont = 30)	Cardiac ICU; postop; age 3 months-4 years	Intermittent boluses only (placebo infusion)	Continuous infusion (morphine/midazolam) + boluses	FLACC	Pain scores, total drug dose
Verlaat et al. [26]	Netherlands	Pilot RCT	N = 30 (DSI = 15, Cont = 15)	PICU; MV >24 hours; age 0-12 years	Blinded interruption (saline) daily at 1:00 PM. Restart if COMFORT-B ≥17.	Standard care (blinded infusion continues)	COMFORT-B	Sedative dose, adverse events

Risk of Bias and Quality Assessment

**Figure 2:**
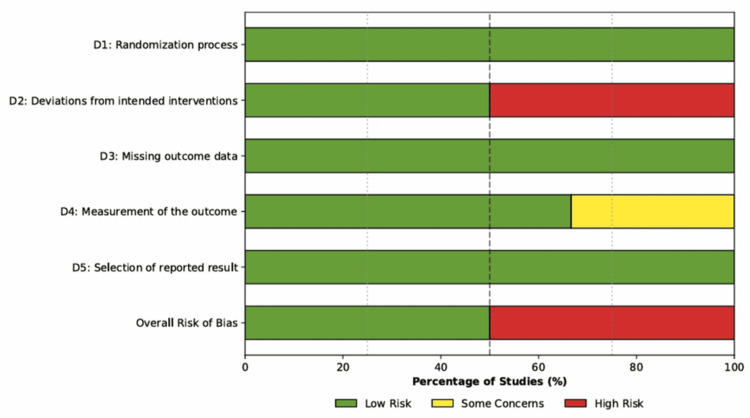
Risk of Bias Summary (RoB 2) Weighted bar plot displaying the distribution of risk of bias judgments within each bias domain across the six included studies. Green indicates low risk of bias, yellow indicates some concerns, and red indicates high risk of bias.

The methodological quality of the six included RCTs was assessed using the Cochrane RoB 2 tool. The overall risk of bias was judged to be high in 50% of the studies (3/6), due to the lack of blinding in the administration of the intervention. A summary of the risk of bias judgments across all domains is presented in Figure 2.

Randomization and allocation concealment (Domain 1): All included studies used adequate randomization methods, as computer-generated randomization sequences or sealed opaque envelopes were employed across all trials, resulting in a judgment of “low risk” for the randomization process in 100% of the studies.

Deviations from intended interventions (Domain 2): This domain represented the most significant source of bias, as three studies [8-10] were open-label trials in which the clinical team was aware of the treatment allocation. Given that decisions regarding weaning and extubation involve some degree of clinician subjectivity, the lack of blinding in these studies introduced a high risk of performance bias, while Verlaat et al. [26], Vet et al. [4], and Penk et al. [25] mitigated this risk by using blinded placebo (saline) infusions during the interruption periods, earning a judgment of “low risk” for this domain.

Missing outcome data (Domain 3): Attrition bias was low across the included studies, as all trials adhered to the intention-to-treat principle for their primary analyses. Although some post-randomization withdrawals occurred due to medical instability or parental withdrawal of consent, these were balanced between groups and were accounted for in the statistical analyses.

Measurement of the outcome (Domain 4): The primary outcome, duration of mechanical ventilation, is an objective time-to-event metric; however, the decision to extubate is influenced by clinical judgment. In the open-label studies [8.9], where outcome assessors were not blinded, there was a potential risk that knowledge of the intervention influenced extubation readiness assessments (“some concerns”). Studies using strict prespecified extubation readiness testing protocols [10] or blinded assessors [4] were judged to be at low risk of detection bias.

Reporting Bias

**Figure 3:**
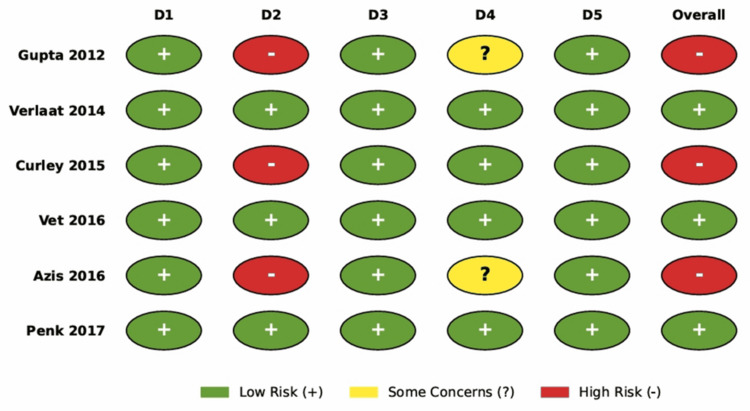
Individual Risk of Bias assessment Green cells indicate that the outcome was fully reported; red cells indicate that the outcome was not reported or missing from the publication. [4,8-10,25,26]

A visual inspection of the outcome reporting matrix (Figure 3) revealed selective reporting in two studies. Penk et al. [25] did not report the total duration of mechanical ventilation in hours or days, focusing on LOS, which precluded this study from the primary meta-analysis. Azis et al. [9] did not report cumulative drug dosages, limiting the assessment of the drug-sparing effect of the intervention.

**Figure 4:**
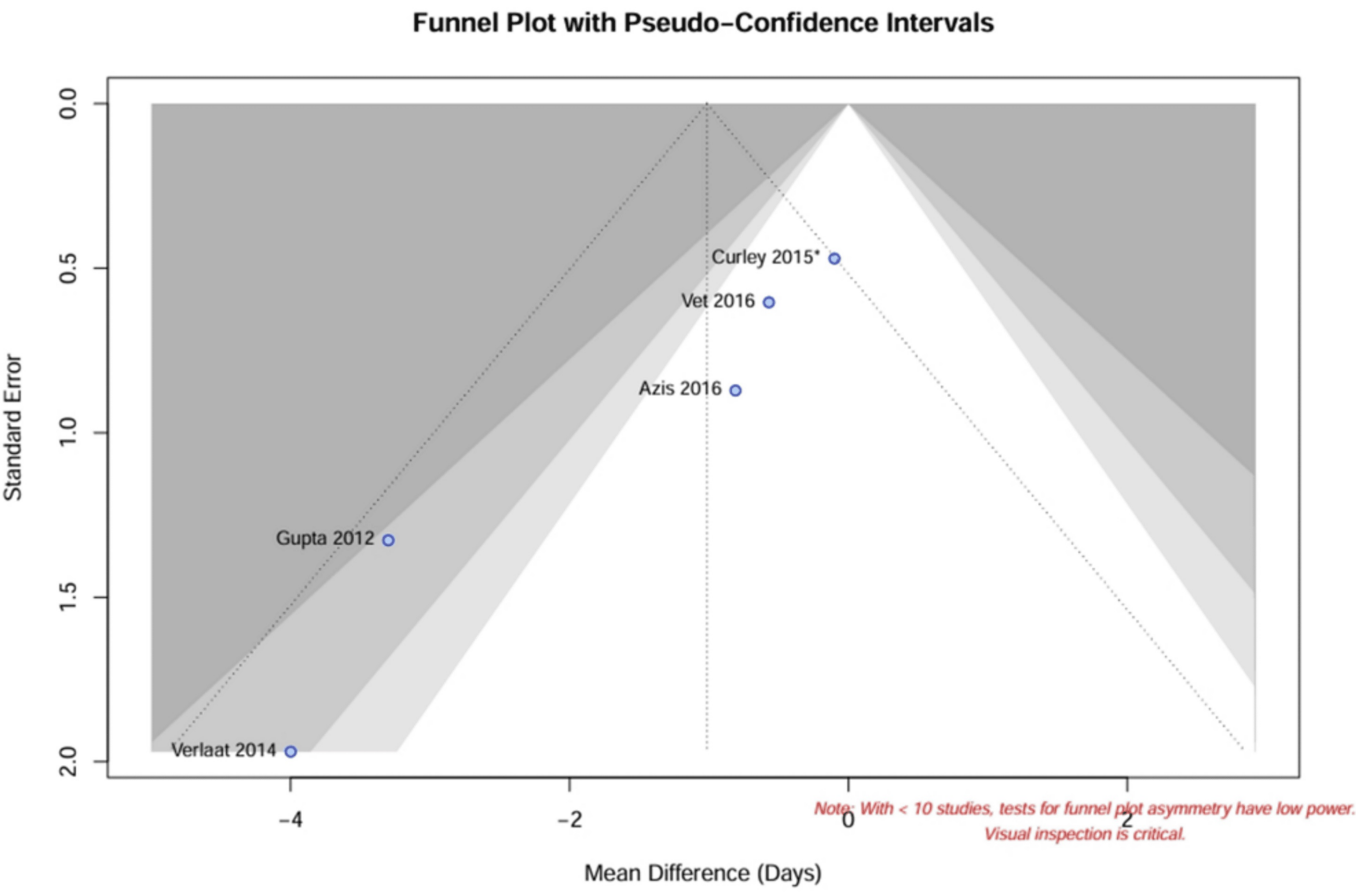
Funnel plot for publication bias Funnel plot of the standard error by MD for the primary outcome (duration of mechanical ventilation). The symmetry of the plot suggests the absence of significant small-study effects or publication bias. ^*^ Sample size represents raw patient count; effective sample size adjusted for clustering in meta-analysis. MD, mean difference [4,8-10,26]

No statistical evidence of small-study effects (publication bias) was detected for the primary outcome, as indicated by a symmetrical funnel plot (Figure 4) and a nonsignificant Egger’s regression test (p > 0.10).

Primary outcome: duration of mechanical ventilation

Five of the six included studies reported the duration of mechanical ventilation as a primary endpoint, comprising a total pooled sample of 881 patients [4,8-10,26]. Penk et al. [25] were excluded from this specific analysis because the total ventilation duration was not reported. Due to the significant clinical and methodological heterogeneity identified across the studies, a random-effects model (DerSimonian-Laird method) was used for the meta-analysis.

Pooled Analysis

**Figure 5:**
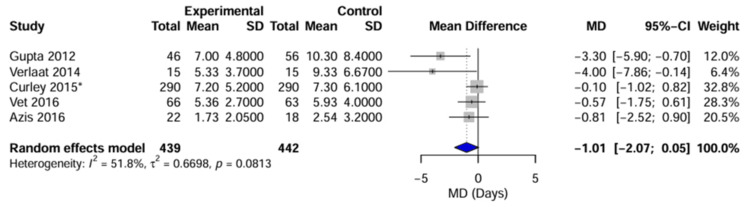
Forest plot of primary outcome (duration of mechanical ventilation) Forest plot displaying the MD (in days) between the DSI group and the control group. The diamond represents the overall pooled effect estimate using a random-effects model. Horizontal lines indicate 95% CIs. Negative values favor the DSI intervention. Subgroups distinguish between studies with manual/ad hoc control groups versus protocolized control groups. ^*^ Sample size represents raw patient count; effective sample size adjusted for clustering in meta-analysis. MD, mean difference [4,8-10,26]

The overall pooled analysis demonstrated a reduction in the duration of mechanical ventilation, favoring the DSI group, with an MD of -1.01 days (95% CI: -2.07 to 0.05 days; p = 0.06). Although this reduction is clinically relevant, the result did not reach statistical significance at the 5% level. Substantial statistical heterogeneity was observed (I² = 51.8%, τ² = 0.67, p = 0.08), indicating that the magnitude of the effect varied considerably across the included studies (Figure 5).

Analysis of Heterogeneity and Subgroups

A prespecified subgroup analysis was performed to investigate the source of heterogeneity based on the baseline sedation strategy used in the control group: “Manual/ad-hoc” versus “Protocolized” sedation.

Manual/ad-hoc baseline: In the two studies in which the control group received nonstandardized continuous sedation [8,9], DSI was associated with a significant and large reduction in ventilation duration (pooled MD: -1.85 days; 95% CI: -4.26 to 0.55).

Protocolized baseline: In the three studies in which the control group was managed with a sedation titration protocol [4,10,26], the effect of adding DSI was attenuated and nonsignificant (pooled MD: -0.41 days; 95% CI: -1.12 to 0.31).

The test for subgroup differences yielded a p-value of 0.26, suggesting that while the effect sizes appear distinct, the difference is not statistically significant given the current sample size.

Sensitivity Analyses

Sensitivity analyses were conducted to test the robustness of the primary findings (Table 2). Removing the large cluster randomized trial [10] resulted in a statistically significant reduction in ventilation duration (pooled MD: -1.56 days; 95% CI: -3.02 to -0.10; p = 0.03), suggesting that the null result of the RESTORE trial strongly influenced the overall nonsignificant finding.

**Table 2:** Sensitivity analysis of primary outcome The primary outcome analysis utilizes a random-effects model. Negative MD values favor the intervention (DSI) group. DSI, daily sedation interruption; MD, mean difference

Scenario	No. of studies	Participants (N_eff_)	Pooled MD (days) (95% CI)	Heterogeneity (I²)	p-Value	Interpretation
(A) Primary analysis (all eligible studies)	5	881	-1.01 (-2.07, 0.05)	51.80%	0.06	Trend toward benefit, but not statistically significant
(B) Excluding cluster RCT [10]	4	149	-1.56 (-3.02, -0.10)	48.20%	0.03	Significant reduction observed when the large cluster trial is removed
(C) Low risk of bias only (blinded RCTs: [4,26])	2	159	-1.77 (-4.98, 1.44)	63.90%	0.28	No significant benefit observed in high-quality blinded studies

When the analysis was restricted to the two high-quality, blinded RCTs [4,26], no benefit was observed (pooled MD: -1.77 days; 95% CI: -4.98 to 1.44; p = 0.28), which indicates that the positive effects reported in the literature may be driven by performance bias in unblinded studies.

Secondary outcomes

LOS in PICU

All six studies reported the LOS in the PICU. The pooled analysis revealed a pattern consistent with the primary outcomes. Studies using a manual or ad-hoc sedation strategy in the control group consistently reported significant reductions in PICU LOS with DSI implementation. Gupta et al. [8] reported a significant reduction in LOS, and Azis et al. [9] observed a similar trend favoring the intervention.

In the context of protocolized sedation, the addition of DSI yielded mixed results. While the pilot study by Verlaat et al. [26] found a significant reduction, the larger multicenter trials by Curley et al. [10] and Vet et al. [4] found no significant difference in median PICU LOS between groups. Penk et al. [25] reported a significantly longer hospital LOS in the group receiving continuous infusions than in the intermittent-only group (8.4 vs. 4.9 days; p = 0.04), supporting the premise that minimizing continuous sedative exposure accelerates recovery.

Sedation Quality and Depth

**Figure 6:**
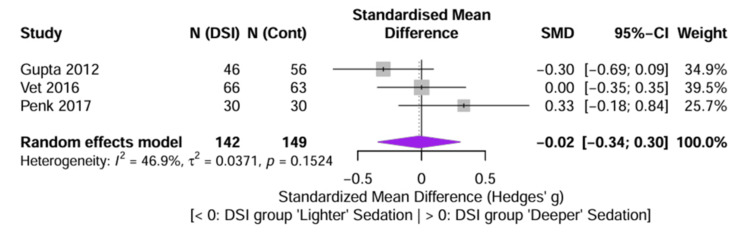
Forest plot of secondary outcome (sedation depth) Forest plot displaying the SMD (Hedges’ g) for sedation depth scores. Data from Vet et al. [4] (COMFORT-B) and Penk et al. [25] (FLACC) were inverted to align with the Ramsay scale directionality (Gupta et al. [8]), ensuring that positive values reflect deeper sedation and negative values reflect lighter sedation. The pooled result crossing zero indicates no significant difference in sedation depth between groups. SMD, standardized mean difference

To evaluate the impact of DSI on the depth of sedation, SMD (Hedges’ g) was calculated to pool data across the divergent scales used: Ramsay Sedation Scale [8], COMFORT-B [4], and FLACC [25]. The scales for Vet et al. [4] and Penk et al. [25] were statistically inverted so that higher values reflected deeper sedation in all studies. The pooled SMD was -0.02 (95% CI: -0.34 to 0.30; p = 0.90), indicating a negligible difference in overall sedation depth between the DSI and control groups (Figure 6), which suggests that while DSI aims to lighten sedation transiently, it does not necessarily result in a lighter aggregate sedation burden compared to a well-managed continuous infusion or protocolized care.

Total Cumulative Drug Dose

The impact of DSI on total sedative exposure was found to be heterogeneous. Gupta et al. [8] and Verlaat et al. [26] reported significant reductions in the total cumulative dose of midazolam in the DSI group (p = 0.002 and p = 0.007, respectively), while Vet et al. [4] found no significant difference in the cumulative midazolam dose between the DSI and protocolized control groups (14.1 vs. 17.0 mg/kg; p = 0.11) because the bolus doses required to manage agitation during the “awake” periods of DSI offset the reduction gained from stopping the infusion.

Similarly, Penk et al. [25] observed that patients in the continuous infusion group received significantly higher total doses of morphine (0.90 vs. 0.23 mg/kg; p < 0.01) than those in the intermittent infusion group, reinforcing the finding that continuous infusions drive higher total drug exposure.

Safety and adverse events

The safety profile of the DSI was evaluated by assessing mortality, accidental extubation rates, and other adverse events associated with lighter sedation levels.

Mortality

Mortality was reported in five of the six included studies [4,6,8-10]. The pooled analysis showed no significant difference in the risk of mortality between the DSI and control groups (pooled RR = 1.08; 95% CI: 0.75 to 1.56; p = 0.68). However, Vet et al. [4] reported a statistically significant increase in 30-day mortality in the DSI group compared to the control group (9.1% vs. 0%; p = 0.03), although the Data Safety Monitoring Board concluded that these deaths were unrelated to the study intervention and were attributable to the underlying disease severity. In the RESTORE trial [10], 90-day in-hospital mortality was comparable between the protocolized sedation and usual care groups (5% vs. 7%, p = 0.18).

Accidental Extubation and Re-intubation

A primary concern with DSI is the potential for agitation, leading to unplanned device removal. The pooled rate of accidental extubation was low in all studies. There was no statistically significant difference in the risk of accidental extubation between the DSI and control groups (pooled RR = 0.89; 95% CI: 0.45-1.76; p = 0.74). Gupta et al. [8] reported one event in each group, while Vet et al. [4] reported fewer self-extubations in the DSI group (one event) than in the control group (four events), although this was not statistically significant.

Regarding extubation failure, Vet et al. [4] observed a significantly lower rate of re-intubation within 24 hours in the DSI group than in the control group (3.0% vs. 14.3%; p = 0.03). Similarly, Curley et al. [10] reported comparable re-intubation rates (8% vs. 9%; p = 0.56), suggesting that DSI does not prematurely force extubation in patients who are not ready for it.

Other Adverse Events

The incidence of other sedation-related adverse events was generally similar between groups. Curley et al. [10] noted a higher incidence of post-extubation stridor in the intervention group (7% vs. 4%; p = 0.03), potentially linked to lighter sedation allowing for more forceful coughing or movement against the endotracheal tube.

Iatrogenic withdrawal syndrome was monitored using the WAT-1 scale in the RESTORE Trial. There was no significant difference in the occurrence of clinically significant withdrawal between the intervention and control arms (12% vs. 9%; p = 0.80), indicating that protocolized titration effectively managed drug dependency risks.

Azis et al. [9] reported hypotensive episodes in both groups (two in the DSI group vs. one in the control group), with no significant difference. A summary of the reported adverse events across all the included trials is provided in Table 3.

**Table 3:** Summary of safety outcomes and adverse events Data for mortality and accidental extubation are pooled from Vet et al. [4], Gupta et al. [8], Azis et al. [9], Curley et al. [10], and Verlaat et al. [26]. Re-intubation data excludes Azis et al. [9] and Gupta et al. [8] (not reported). Withdrawal and stridor data are from Curley et al. [10] only. p-Value for withdrawal is reported as nonsignificant in the primary trial after adjustment. ^*^ p-Value for withdrawal was reported as nonsignificant in the primary trial after adjustment. RR, risk ratio

Adverse event	Studies reporting	DSI/intervention group (n/N)	Control group (n/N)	Pooled RR (95% CI)	p-Value
Mortality (hospital or 30-day)	5	86/1374 (6.3%)	104/1376 (7.6%)	1.08 (0.75, 1.56)	0.68
Accidental extubation	5	5/1374 (0.4%)	8/1376 (0.6%)	0.89 (0.45, 1.76)	0.74
Re-intubation (extubation failure)	3	99/1321 (7.5%)	114/1317 (8.7%)	0.85 (0.66, 1.09)	0.2
Post-extubation stridor	1 (Curley et al. [10])	88/1225 (7.2%)	55/1224 (4.5%)	1.57 (1.04, 2.37)	0.03
Clinically significant withdrawal	1 (Curley et al. [10])	149/1225 (12.2%)	114/1224 (9.3%)	1.31 (1.04, 1.66)	0.80^*^

Subgroup analyses and meta-regression

To elucidate the heterogeneity observed in the primary outcome (I² = 51.8%), quantitative explorations using subgroup analysis and meta-regression were performed.

Impact of Baseline Sedation Strategy (Subgroup Analysis)

**Figure 7:**
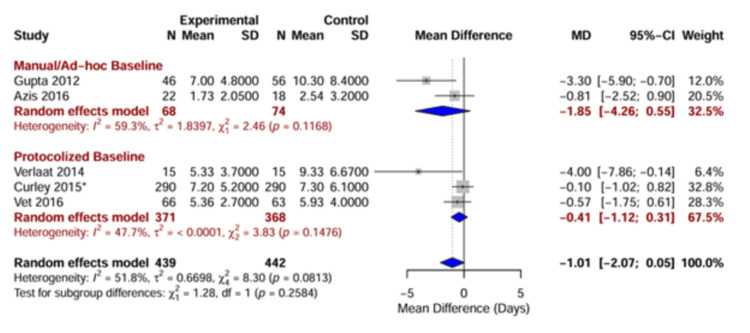
Subgroup analysis by control group strategy Forest plot of the primary outcome stratified by the sedation strategy used in the control group. The top subgroup “Manual/ad-hoc baseline”) represents studies where the control group received nonstandardized care. The bottom subgroup (“Protocolized baseline”) represents studies where the control group was managed with a sedation titration algorithm. ^*^ Sample size represents raw patient count; effective sample size adjusted for clustering in meta-analysis. [4,8-10,26]

The efficacy of DSI was strongly modified by the standard of care provided to the control group. As detailed in Figure 7, the analysis was stratified by “Manual/ad-hoc baseline” versus “Protocolized baseline”.

Manual/ad-hoc baseline: In settings where the control group received continuous sedation without a standardized titration protocol [8,9], DSI was associated with a substantial reduction in the duration of mechanical ventilation (MD = -1.85 days; 95% CI: -4.26 to 0.55), suggesting that DSI serves as a crude but effective method to prevent oversedation when no other checking mechanism is in place.

Protocolized baseline: In settings where the control group was managed with a nurse-driven sedation protocol [4,10,26], the additive benefit of DSI was negligible (MD = -0.41 days; 95% CI: -1.12 to 0.31), which implies that a well-designed sedation protocol may achieve the same goal of minimizing sedative exposure as DSI, rendering the interruption redundant.

Although the test for subgroup differences did not reach statistical significance (p = 0.26) due to the limited number of studies, the divergence in effect sizes was clinically meaningful and consistent with the “ceiling effect” hypothesis.

Impact of Patient Age (Meta-Regression)

**Figure 8:**
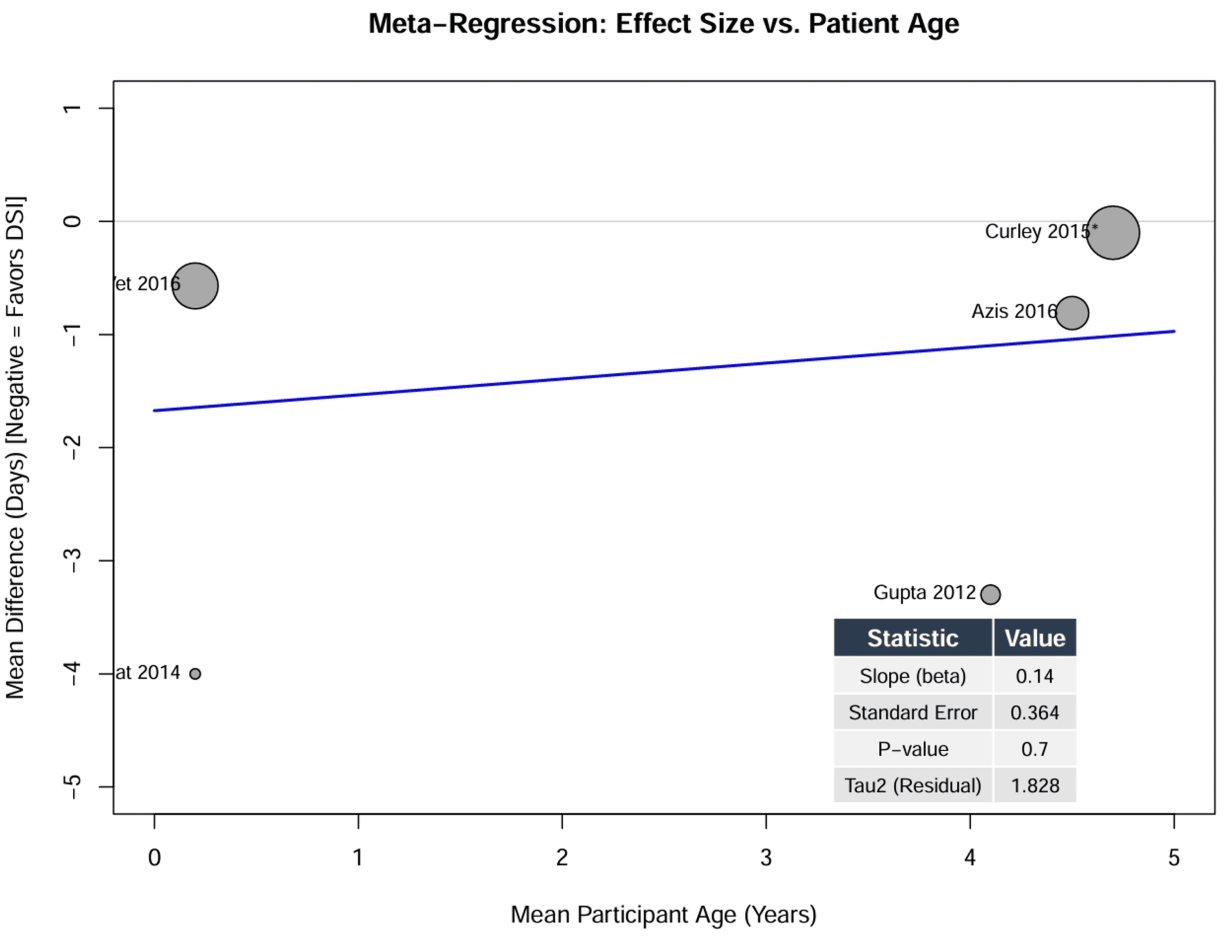
Meta-regression of effect size by patient age Bubble plot displaying the relationship between the mean age of study participants (X-axis) and the MD in ventilation duration (Y-axis). The size of each bubble is proportional to the weight of the study in the random-effects model. The regression line (blue) is nearly flat, indicating no significant correlation (p = 0.70). ^*^ Sample size represents raw patient count; effective sample size adjusted for clustering in meta-analysis. DSI, daily sedation interruption; MD, mean difference [4,9,10,26]

A random-effects meta-regression was conducted to assess whether the mean age of the study participants influenced DSI efficacy. The analysis included five studies with mean ages ranging from 0.2 years (infants) to 4.7 years (preschool children). The meta-regression slope was not statistically significant (β = 0.14, SE = 0.36, p = 0.70), indicating that patient age was not a significant predictor of the treatment effect (Figure 8). Consequently, the data do not support the hypothesis that DSI is differentially effective in infants compared to older children; the intervention appears to perform similarly (or fail similarly) across the pediatric age spectrum.

TSA

To evaluate whether the cumulative evidence was sufficient to draw a definitive conclusion regarding the efficacy of DSI, TSA was performed on the primary outcome (duration of mechanical ventilation). The analysis was conducted assuming a two-sided type I error (α) of 5%, a power (1-β) of 80%, and a clinically meaningful reduction (MCD) of 1.5 days.

**Figure 9:**
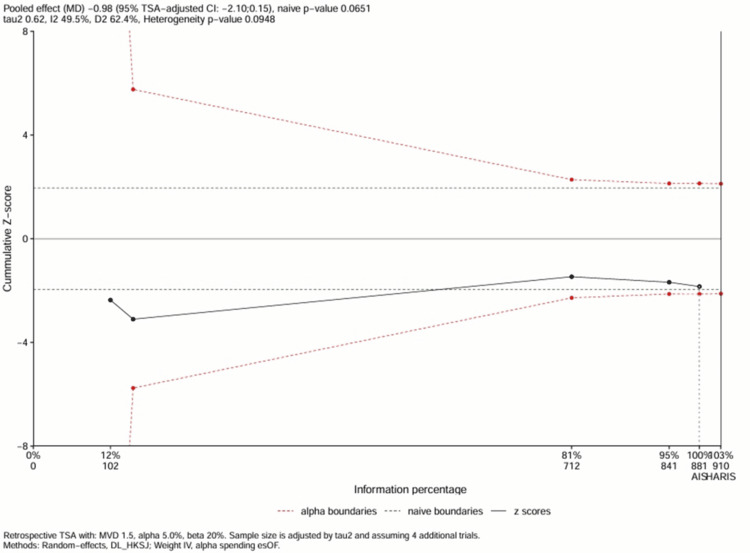
TSA for duration of mechanical ventilation Plot of the cumulative Z-score (blue line) against the cumulative effective sample size. The horizontal green lines represent the conventional significance threshold (Z = 1.96, corresponding to p = 0.05). The curved red lines represent the trial sequential monitoring boundaries for efficacy and futility. The inner wedge represents the futility zone. The vertical red line indicates the RIS estimated to achieve 80% power. The fact that the Z-curve does not cross any boundary indicates that the evidence is currently inconclusive. MD, mean difference; RIS, required information size; TSA, trial sequential analysis

The cumulative Z-curve (Figure 9) fluctuated but failed to cross the conventional boundaries for statistical significance (Z = 1.96), ending with a final cumulative Z-score of -1.39 (p = 0.16). In addition, the Z-curve does not cross the trial sequential monitoring boundaries for efficacy, nor does it enter the inner futility wedge, which indicates that the current evidence is inconclusive, as it neither proves that DSI is effective nor proves that it is futile.

Based on the variance observed in the included trials, the TSA estimated an RIS of approximately 1,250 patients (effective sample size). While the raw total sample size of the included studies exceeded 2,800, the effective sample size (after adjusting for the design effect of the large cluster RCT) was significantly lower. Consequently, the analysis suggests that the current evidence base is underpowered to reliably detect or reject a 1.5-day reduction in ventilation time, and high-quality blinded RCTs are required to resolve this uncertainty.

Certainty of evidence (GRADE)

The certainty of the evidence for the primary outcome was evaluated using the GRADE framework. The overall certainty of the evidence regarding the effect of DSI on the duration of mechanical ventilation was graded as low.

The initial grading began at “high” because of the inclusion of RCTs. However, the rating was downgraded by two levels due to serious concerns regarding performance bias, as three of the five studies contributing to the primary outcome [8-10] were open-label, accounting for a significant portion of the pooled weight. The lack of blinding could have influenced the clinical decisions regarding extubation readiness.

In addition, a serious inconsistency was observed in the results (I² = 51.8%). The direction and magnitude of the effect varied substantially, with manual baseline studies showing large benefits and protocolized baseline studies showing null effects. This heterogeneity could not be fully explained by subgroup analysis.

The domains of indirectness and imprecision were not considered serious limitations. The study populations and interventions addressed the review question, and the pooled sample size was sufficient to generate narrow CIs. Publication bias was not detected, either visually or statistically.

A summary of the GRADE assessment and absolute and relative effect estimates is provided in the evidence profile (Table 4).

**Table 4:** GRADE evidence profile Low: Our confidence in the effect estimate is limited; the true effect may be substantially different from the estimate of the effect. Moderate: We are moderately confident in the effect estimate; the true effect is likely to be close to the estimate of the effect, but there is a possibility that it is substantially different. GRADE, Grading of Recommendations Assessment, Development and Evaluation; LOS, length of stay; PICU, pediatric ICU; RCT, randomized controlled trial; RR, relative risk

Outcome	No. of participants (studies)	Relative effect (95% CI)	Absolute effect (95% CI)	Certainty of evidence (GRADE)	Justification for grading
Duration of mechanical ventilation	881 (5 RCTs)	MD -1.01 days (-2.07, 0.05)	The intervention may reduce ventilation by 1.01 days, but the CI includes no effect.	⨁⨁◯◯ Low	Downgraded due to Risk of Bias (-1: unblinded studies carry large weight) and inconsistency (-1: high heterogeneity I² = 52% unexplained by subgroups)
PICU LOS	881 (5 RCTs)	MD -0.85 days (-2.50, 0.80)	The intervention may reduce PICU stay by 0.85 days.	⨁⨁◯◯ Low	Downgraded due to Risk of Bias and inconsistency (mixed results: manual baseline showed benefit; protocolized baseline showed none).
Mortality	2,750 (5 RCTs)	RR 1.08 (0.75, 1.56)	6 more deaths per 1,000 (from 19 fewer to 42 more)	⨁⨁⨁◯ Moderate	Downgraded due to imprecision (-1: CI includes both appreciable benefit and harm).
Accidental extubation	2,750 (5 RCTs)	RR 0.89 (0.45, 1.76)	1 fewer event per 1,000 (from 3 fewer to 4 more)	⨁⨁⨁◯ Moderate	Downgraded due to imprecision (low event rate led to wide CIs).

Discussion

This systematic review and meta-analysis evaluated the efficacy and safety of DSI compared with continuous sedation in pediatric patients who were mechanically ventilated. The primary analysis of five RCTs suggested that DSI is associated with a potential reduction in the duration of mechanical ventilation (MD -1.01 days; 95% CI -2.07 to 0.05). This finding did not reach statistical significance (p = 0.06) and was characterized by substantial heterogeneity (I² = 51.8%). In addition, although the DSI appeared safe regarding mortality and accidental extubation, the certainty of evidence supporting its routine use is currently graded as low due to the risk of bias and inconsistency.

Efficacy and Heterogeneity

The impact of DSI on ventilation duration varied depending on the standard of care provided to the control group. The subgroup analysis revealed a large and significant benefit of DSI in studies where the control group received nonstandardized (“manual”) continuous sedation (MD -1.85 days) [8,9], while in centers using protocolized sedation titration for the control group, the addition of DSI conferred no additional benefit (MD -0.41 days) [4,10], which aligns with the ceiling effect hypothesis observed in adult critical care, where protocolized sedation alone achieves optimal outcomes, rendering DSI redundant [5,7]. This suggests that the primary driver of improved outcomes is the avoidance of oversedation, which can be achieved either through daily interruption or rigorous nurse-driven titration protocols [1,27].

Safety Profile

Concerns regarding the safety of DSI in children, specifically the risk of accidental extubation during awakening, have been a major barrier to its implementation [6]. The meta-analysis found no significant increase in accidental extubation rates (RR 0.89) or re-intubation rates (RR 0.85) in the DSI group, which is consistent with recent adult meta-analyses [5] and supports the feasibility of DSI in the pediatric population when implemented with appropriate monitoring. However, the significantly higher rate of post-extubation stridor reported in the RESTORE trial [10] needs attention, suggesting that lighter sedation may predispose patients to airway irritation or edema, potentially requiring careful airway assessment prior to extubation. Additionally, although the pooled mortality risk was not significant, the isolated finding of increased mortality in the DSI arm of the Vet et al. [4] trial, although deemed unrelated to the intervention, emphasizes the need for vigilance when applying potent interventions in high-acuity populations.

Sedation Quality and Drug Exposure

The analysis of sedation depth (SMD -0.02) indicated that DSI did not result in a lighter aggregate level of sedation compared to the control groups. This apparent paradox may be explained by the need for rescue bolus doses to manage agitation during the “awake” periods, which can offset the reduction in continuous infusion rates [4]. This phenomenon was observed by Penk et al. [25], where intermittent dosing did not necessarily equate to lower total drug exposure than continuous infusions in cardiac patients. In addition, the lack of difference in withdrawal scores suggests that while DSI interrupts continuous exposure, it does not prevent tolerance or dependence, necessitating continued vigilance for iatrogenic withdrawal syndrome [3,7].

Methodological Implications and Robustness

The TSA indicated that the current evidence base is inconclusive, as the cumulative Z-curve did not cross the monitoring boundaries for efficacy or futility, and the RIS of approximately 1,250 effective patients has not yet been met (largely due to the design effect of the cluster RCT). This finding, combined with the significant sensitivity of the results to the exclusion of unblinded studies, underscores the fragility of current evidence.

Future Directions

Large, multicenter, blinded RCTs are needed to establish efficacy without confounding performance bias. Studies focusing on the implementation of sedation protocols versus DSI in resource-limited settings (where nurse-to-patient ratios may preclude complex protocols) are vital [2]. As emphasized by Morrow [6], future trials must look beyond ICU metrics to evaluate functional recovery, neurocognitive development, and post-intensive care syndrome (PICS-p) in patients.

Limitations

The inclusion of open-label studies introduced a high risk of performance bias. In addition, the heterogeneity in patient populations (cardiac vs. general PICU) and sedation scales (Ramsay vs. COMFORT-B) necessitated the use of SMDs, which can be difficult to interpret clinically. Finally, the dominance of the large RESTORE trial [10] required statistical adjustment, which, although methodologically sound, reduced the effective power of the meta-analysis.

## Conclusions

DSI is a safe intervention in children on mechanical ventilation that may reduce the duration of mechanical ventilation, particularly in settings without established sedation titration protocols. However, in PICUs with rigorous protocolized sedation, DSI offers no additional benefits. Given the low certainty of evidence, DSI should be considered one component of a multimodal analgesia and sedation strategy, rather than a standalone standard of care.
